# Magnetic Resonance Imaging Evaluation of Functional Differences Between In Vivo Human Kidneys and Discarded Human Kidneys During Ex Vivo Normothermic Machine Perfusion

**DOI:** 10.1111/aor.70142

**Published:** 2026-04-27

**Authors:** Tim L. Hamelink, Chris L. Jaynes, L. Annick van Furth, Johannes Castelein, Nathan Ooms, Veerle A. Lantinga, Baran Ogurlu, Xiaopeng Zhou, Ulrike Dydak, Henri G. D. Leuvenink, Anna Krarup Keller, Ronald J. H. Borra, Cyril Moers

**Affiliations:** ^1^ Department of Surgery, Organ Donation and Transplantation University Medical Center Groningen, University of Groningen Groningen the Netherlands; ^2^ 34 Lives, Public Benefit Corporation West‐Lafayette Indiana USA; ^3^ Department of Radiology University Medical Center Groningen Groningen the Netherlands; ^4^ Department of Biomedical Sciences Faculty of Health and Medical Sciences, University of Copenhagen Copenhagen Denmark; ^5^ School of Health Sciences, Purdue University West‐Lafayette Indiana USA; ^6^ Department of Urology Aarhus University Hospital Aarhus Denmark; ^7^ Department of Clinical Medicine Aarhus University Aarhus Denmark

**Keywords:** kidney transplantation, magnetic resonance imaging, normothermic machine perfusion, viability assessment

## Abstract

**Background:**

With increasing interest in renal normothermic machine perfusion (NMP), a deeper understanding of ex vivo renal physiology is essential to establish NMP as a robust pre‐transplant assessment platform. This study utilized magnetic resonance imaging (MRI) to compare in vivo renal physiology in healthy volunteers with ex vivo renal function of human donor kidneys during NMP.

**Methods:**

Multiparametric MRI maps assessing water diffusion, oxygenation, tissue characteristics, and perfusion were obtained from 11 healthy volunteers to define an in vivo renal reference frame. For ex vivo evaluation, 25 discarded human donor kidneys underwent 4 h of oxygenated hypothermic machine perfusion, followed by 6 h of NMP in an MRI‐compatible setup. The kidneys were assessed hourly using comparable MRI modalities as in vivo.

**Results:**

Ex vivo renal physiology was substantially different from in vivo physiology in terms of MRI‐based diffusion patterns, oxygenation, tissue characteristics, and tissue perfusion. Most MRI measurements did not correlate well with conventional parameters such as flow, renal function, and injury markers during NMP.

**Conclusions:**

Our findings highlight distinct differences between in vivo and ex vivo MRI‐based renal characteristics in a human cohort, suggesting that parameters beyond conventional in vivo functional markers may warrant consideration when evaluating organ viability during NMP.

## Graphical abstract

Comparative MRI of in vivo kidneys and ex vivo donor kidneys during NMP revealed marked physiological differences and limited correspondence with conventional perfusion or injury metrics. These distinct ex vivo MRI signatures emphasize the need to consider parameters beyond conventional in vivo functional markers when assessing organ viability during NMP.

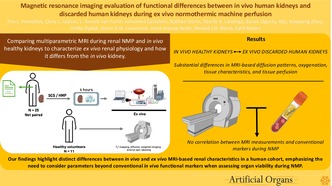

**Abbreviations**
ADC: apparent diffusion coefficient
ASL: arterial spin labeling
ATP: adenosine triphosphate
BOLD: blood oxygen level‐dependent
CIT: cold ischemia time
CMD: corticomedullary difference
CrCl: creatinine clearance
DBD: donation after brain death
DCD: donation after circulatory death
DWI: diffusion‐weighted imaging
FENa^+^
: fractional sodium excretion
HMP: hypothermic machine perfusion
HMPO_2_
: oxygenated hypothermic machine perfusion
KDPI: kidney donor profile index
MAP: mean arterial pressure
NMP: normothermic machine perfusion
OPOs: Organ Procurement Organizations
pCASL: pseudo‐continuous arterial spin labeling
RBC: red blood cell
RBF: renal blood flow
SCS: static cold storage
VO_2_
: oxygen consumption
WIT: warm ischemic time

## 1 Introduction

In response to the growing demand for donor kidneys, there has been an increased reliance on kidneys from donors of advanced age and with multiple comorbidities, contributing to higher rates of kidney discard. Discard rates of deceased‐donor kidneys across Europe approximate 10%–12%, while discard rates in the United States soared to almost 29% in recent years [1, 2]. This discard rate, in combination with considerable variability in the decision‐making process among transplant specialists [3], fuels the interest in more refined organ assessment strategies. Interest in normothermic machine perfusion (NMP) as a promising technique to assess organ quality prior to transplantation is on the rise [4, 5]. The (re)evaluation of initially discarded deceased human donor kidneys during NMP holds the potential to reintroduce a portion of these organs into the donor pool. However, despite the proven safety and feasibility of clinical NMP [6, 7, 8], the field still lacks robust ex vivo perfusion‐associated biomarkers that can reliably distinguish kidneys suitable for transplantation from those rightfully declined. A further challenge is that interpretation of NMP‐derived readouts is often benchmarked against the in vivo reference frame. However, ex vivo perfusion represents a fundamentally different physiologic state, particularly in marginal donor kidneys subjected to prolonged (cold) ischemia. To address this, we applied multiparametric magnetic resonance imaging (MRI) during both renal NMP of discarded human kidneys and in vivo kidneys from healthy volunteers to better understand ex vivo renal physiology and to characterize its differences from the in vivo state. Clarifying this “apples‐to‐pears” comparison is essential to avoid misinterpretation of ex vivo renal characteristics and to establish a more appropriate framework for evaluating kidney physiology during NMP. Finally, we examined associations between donor characteristics, NMP parameters, and quantitative MRI features to gain further insights into the complex dynamics of renal function during ex vivo perfusion.

## 2 Materials and Methods

### 2.1 Donor Kidneys and Ethical Approval

Between May 2023 and August 2023, 25 human kidneys from the United Network for Organ Sharing program, deemed unsuitable for transplantation, were included in this study. Kidneys discarded for transplantation were offered by non‐profit Organ Procurement Organizations (OPOs). Consent to use these donor kidneys for research purposes was acquired by the OPOs in adherence to standards outlined in the Uniform Anatomical Gift Act of the United States. The Institutional Review Board of Purdue University granted approval for the in vivo MRI assessment of kidneys from healthy human volunteers (reference number: IRB‐2023‐1807).

### 2.2 Kidney Procurement, Donor Information, and Exclusion Criteria

Standard surgical procedures were employed for kidney retrieval at the OPOs. Following aortic cannulation, kidneys from donation after circulatory death (DCD) and donation after brain death (DBD) donors underwent cold in situ perfusion using University of Wisconsin Cold Storage Solution. After kidney retrieval, they were either placed on hypothermic machine perfusion (HMP) (LifePort Kidney Transporter, Organ Recovery Systems, Itasca, IL, USA) or preserved using static cold storage (SCS) and transported to the laboratory facility once they were declined for transplantation.

Donor information was documented which included donor age, sex, kidney donor profile index (KDPI), donation pathway (DCD/DBD), reason for discard, renal function in the donor, warm ischemic time (WIT), cold ischemia time (CIT), and histological assessment.

All human kidneys offered for research were considered for inclusion and only excluded in case of complex arterial anatomy, structural damage, or a positive donor serology for hepatitis A/B/C or human immunodeficiency virus.

### 2.3 Preparation of Kidneys and Oxygenated Hypothermic Machine Perfusion

After arrival at the research facility, cold preserved kidneys were prepared for a subsequent 4‐h period of oxygenated hypothermic machine perfusion (HMPO_2_). The renal artery was (re)cannulated, the ureter was cannulated with an 8Ch feeding tube (Nutrisafe 2, Vygon, Valkenswaard, the Netherlands), and a 10Ch feeding tube (Vygon) was sutured to the interior venous wall to facilitate direct venous sampling. Hereafter, kidneys were placed on a custom‐built HMPO_2_ system and perfused with University of Wisconsin Machine Perfusion Solution at a pressure of 25 mmHg.

### 2.4 Normothermic Machine Perfusion

**FIGURE 1:**
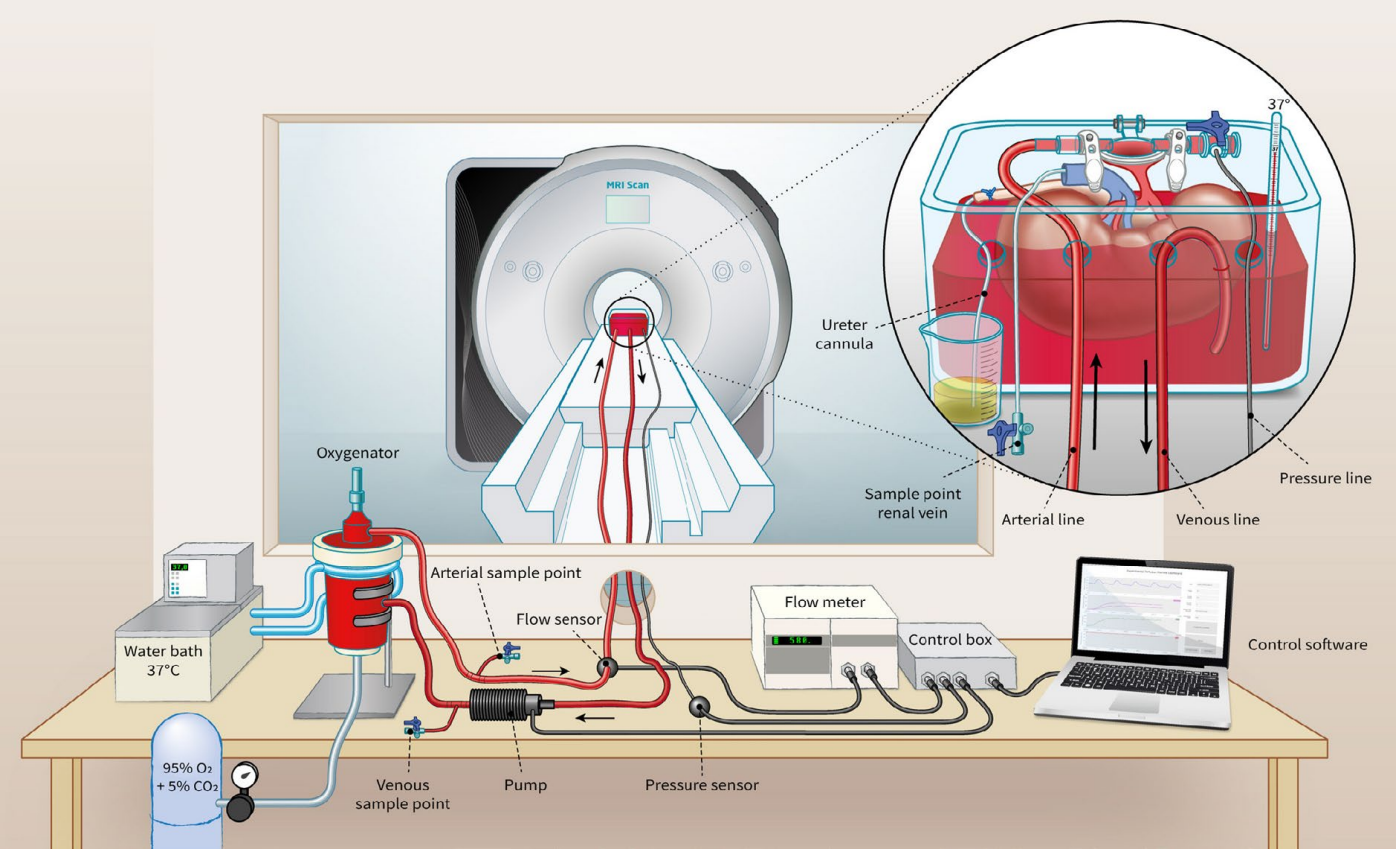
Schematic representation of MRI‐compatible NMP setup. The MRI‐compatible NMP setup comprised a centrifugal pump driven by a pump unit that was controlled by custom‐built hardware and software, an oxygenator/heat exchanger, and blood flow, pressure, and temperature sensors. Seven meters of arterial and venous tubing linked the organ chamber within the MRI's isocenter to the hardware in the control room of the MRI scanner. The image was reprinted from Schutter et al. [9] with permission from the publisher. [Color figure can be viewed at wileyonlinelibrary.com]

Following HMPO_2_, kidneys were connected to an MRI‐compatible NMP setup which was similar to those described earlier by our group and perfused for 6 h (Figure 1) [9, 10, 11, 12]. A detailed description can be found in Supporting Information S1.

### 2.5 Perfusate, Urine, and Tissue Measurements

A detailed description of the sampling procedures and biochemical analyses can be found in Supporting Information S2.

### 2.6 Magnetic Resonance Imaging

A Siemens Prisma 3 T MRI system located at the Purdue MRI Facility in West Lafayette, Indiana was utilized in the in vivo and ex vivo renal assessment (Magnetom Prisma, Siemens Healthineers, Erlangen, Germany). For in vivo kidney imaging, 11 healthy human volunteers aged 40–60 years were positioned in the MRI scanner. This specific age range was selected to match the donor ages of the 25 discarded human donor kidneys we received. An 18‐channel body phased‐array surface coil was positioned on the abdomen, and a multiparametric MRI protocol sequences, including DWI, BOLD/T_2_*‐mapping, T_1_ mapping, and pseudo‐continuous ASL (pCASL), was applied (Supporting Information S3). Moreover, detailed anatomical T_2_‐weighted images were obtained to identify anatomical anomalies and for regional segmentation purposes. For the ex vivo assessment, the organ chamber was placed within a 64‐channel head coil to acquire the same MRI sequences on an hourly basis during 6 h of warm ex vivo perfusion. The raw data from DWI, T_2_* maps, and T_1_ maps underwent post‐processing using nordicICE (version 4.2.0, Nordic Neuro Lab, Bergen, Norway), while Bayesian Inference for Arterial Spin Labeling (BASIL) [13] was used for processing raw pCASL data. Subsequently, the post‐processed data, including quantitative maps reflecting the Brownian motion of water molecules (ADC), tissue characterization (T_2_*/T_1_ mapping), and renal tissue perfusion (ASL), were visualized using Horos (version 4.0.0, Horos Project).

To ensure unbiased analysis, quantitative MRI maps underwent randomization and data analysis was conducted under blinded conditions. The quantitative maps were then combined with the anatomical image to allow segmentation of the renal cortex and medulla. To characterize the relative differences of the renal cortex and medulla, a corticomedullary (CM) ratio was computed for each quantitative MRI parameter.

### 2.7 Statistical Analyses

GraphPad Prism (version 10.2.2, GraphPad Software, Boston, Massachusetts USA) and R (version 4.3.1, R Foundation for Statistical Computing, Vienna, Austria) were used for statistical analyses and data visualization. Data normality was assessed using Q‐Q plots. Key donor characteristics (e.g., KDPI, CIT, donor type, donor age, and terminal serum creatinine of the donor) were divided into groups based on median or literature‐based cutoff criteria, and the differences in quantitative MRI data between these groups were compared using a Mann–Whitney *U* test. The association between the means of conventional NMP parameters and quantitative MRI data was assessed with a generalized linear model using the *stats* package. First, the selection of model distribution (gaussian or gamma) and associated link function were evaluated using a Cullen and Frey plot. Subsequently, a natural cubic spline with three knots was constructed for all models utilizing the *splines* package. Since KDPI already incorporates most donor characteristics, the generalized linear models were adjusted for the KDPI. To account for multiple testing, *p* values were adjusted using the Benjamini–Hochberg false discovery rate procedure. Corrections were applied separately for each group of related analyses, including comparisons between ex vivo and in vivo measurements, associations between MRI parameters and NMP variables, and donor‐related subgroup analyses.

## 3 Results

### 3.1 Characteristics of Donors and Healthy Volunteers

In total, 25 deceased‐donor kidneys were included in this study (Table 1). The donors had a median age of 60 years (IQR 48–66), of which 10 were derived from female donors (40%) and 15 from male donors (60%). Eight kidneys were retrieved from DCD donors (32%) and the remaining 17 from DBD donors (68%). The DCD kidneys had a median WIT of 11 min (IQR 9–13.5), and all kidneys together had a median CIT of 29.5 h (25.4–37.1). The kidneys had a relatively high median KDPI score of 88 (IQR 65.5–94.5) and a median glomerulosclerosis score of 10% (IQR 0–15). The median age of healthy volunteers was 52 years (IQR 44.5–54), with six females and five males. To improve accessibility for non‐radiologists we provide a clear overview of the imaging results to summarize each MRI parameter, its key findings, and potential relevance during normothermic machine perfusion (Table 2).

**TABLE 1:** Donor characteristics.

	Age (y)	Sex	BMI (kg/m^2^)	Donor type	WIT (min)	Terminal serum creatinine (μmol/L)	CIT^b^ (h:min)	KDPI	Glomerulosclerosis score (%)	Reason for discard
	60 [48–66]		28 [23.2–36.4]			156 [78–370]	29.5 [25.4–37.1]	88 [65.5–94.5]	10 [0–15]	
K1	60	Female	43.3	DCD	7	47	31:02	90	0	High KDPI
K2	35	Male	26.5	DBD	n/a	690	19:38	54	0	Poor function in donor
K3	45	Male	40.4	DBD	n/a	359	28:29	89	20	High KDPI
K4	68	Female	25.2	DBD	n/a	55	27:42	88	15	High KDPI
K5	60	Male	26.4	DCD	10	644	28:51	95	10	Poor function in donor
K6	52	Male	31	DBD	n/a	548	33:35	82	6	Poor function in donor
K7	54	Female	38.6	DCD	11	69	45:09	84	68	Biopsy findings
K8	75	Female	18.9	DBD	n/a	80	13:50	98	^a^	High KDPI
K9	72	Female	36.5	DBD	n/a	76	23:04	95	12	High KDPI
K10	65	Male	28	DCD	8	115	24:15	94	13	High KDPI
K11	67	Male	22.2	DBD	n/a	97	14:31	97	46	Biopsy findings
K12	53	Female	23.8	DCD	13	147	29:47	77	^a^	Prolonged period of hypotension in agonal phase
K13	69	Male	33.6	DBD	n/a	557	31:57	89	0	High KDPI
K14	47	Male	30	DBD	n/a	306	37:03	78	20	Poor function in donor
K15	58	Male	31	DBD	n/a	156	28:39	87	0	^a^
K16	49	Female	20.5	DBD	n/a	159	29:49	54	35	Biopsy findings
K17	66	Male	37.9	DCD	14	68	32:07	95	0	High KDPI
K18	32	Male	42	DBD	n/a	310	26:43	20	0	Biopsy findings (ATN)
K19	61	Female	22.5	DCD	19	43	42:51	21	6	High RR during HMP
K20	60	Male	22.3	DCD	11	87	37:12	89	10	Long functional WIT
K21	64	Male	33.4	DBD	n/a	203	43:51	93	13	High KDPI
K22	60	Female	26.2	DBD	n/a	235	39:36	85	6	Biopsy findings
K23	65	Male	27.1	DBD	n/a	690	24:28	97	12	High KDPI
K24	44	Male	36.2	DCD	10	380	34:40	50	10	Long CIT
K25	36	Female	21.8	DCD	6	108	49:20	38	4	Long CIT

*Note:* Data are represented as median and interquartile range.

Abbreviations: ATN, acute tubular necrosis; CIT, cold ischemia time; DBD, donation after brain death; DCD, donation after circulatory death; HMP, hypothermic machine perfusion; KDPI, kidney donor profile index; n/a, not applicable; RR, renal vascular resistance; WIT, warm ischemia time.

^a^ Missing.

^b^ Time from donor nephrectomy until the start of normothermic machine perfusion.

**TABLE 2:** Summary of MRI parameters, key findings, and potential relevance during normothermic machine perfusion.

MRI parameter	Physiological meaning	Key findings (ex vivo vs. in vivo)	Associations with NMP and donor parameters	Potential relevance for NMP assessment
Diffusion‐weighted imaging (ADC)	Reflects the Brownian motion of water molecules; lower values indicate increased diffusion restriction (e.g., edema, fibrosis, cellular injury)	Cortical ADC values were significantly lower in ex vivo kidneys compared to in vivo kidneys. Medullary ADC values were comparable. Corticomedullary difference (CMD) was reduced ex vivo	Positive association between cortical ADC and creatinine clearance during NMP. Lower cortical ADC was observed in kidneys from donors with higher terminal serum creatinine	Could reflect structural integrity and functional capacity during NMP. Potential marker of diffusion restriction and injury
T2* mapping (BOLD‐MRI)	Reflects regional oxygen bioavailability; higher T2* suggests increased oxygen availability or reduced oxygen utilization	Both cortical and medullary T2* values were higher ex vivo than in vivo. Medullary T2* particularly elevated during NMP. Lower T2* observed in kidneys with prolonged cold ischemia time	No consistent associations with renal blood flow, oxygen consumption, or ATP content during NMP	May reflect altered oxygen utilization during NMP. Interpretation is complex due to limited direct overlap with conventional oxygen kinetics measures
T1 mapping	Reflects tissue characteristics including water content, inflammation, and fibrosis	Cortical and medullary T1 values ex vivo were comparable to in vivo measurements. CMD was reduced ex vivo. Considerable variability among ex vivo kidneys	Inverse association between cortical T1 and renal blood flow. No consistent associations with other NMP functional parameters	Could indicate tissue water content and structural variability during NMP. Role in viability assessment during NMP remains exploratory
Arterial spin labeling (ASL)	Measures tissue perfusion by magnetically labeling inflowing blood/perfusate	Cortical perfusion highly variable ex vivo and significantly higher than in vivo. Medullary perfusion was comparable. CM perfusion ratio highly variable ex vivo	No consistent association between ASL‐derived perfusion and total measured renal blood flow	May provide regional perfusion information beyond total flow measurements. However, interpretation is difficult due to highly variable flow dynamics during NMP

### 3.2 In Vivo and Ex Vivo Magnetic Resonance Imaging Assessment

**FIGURE 2:**
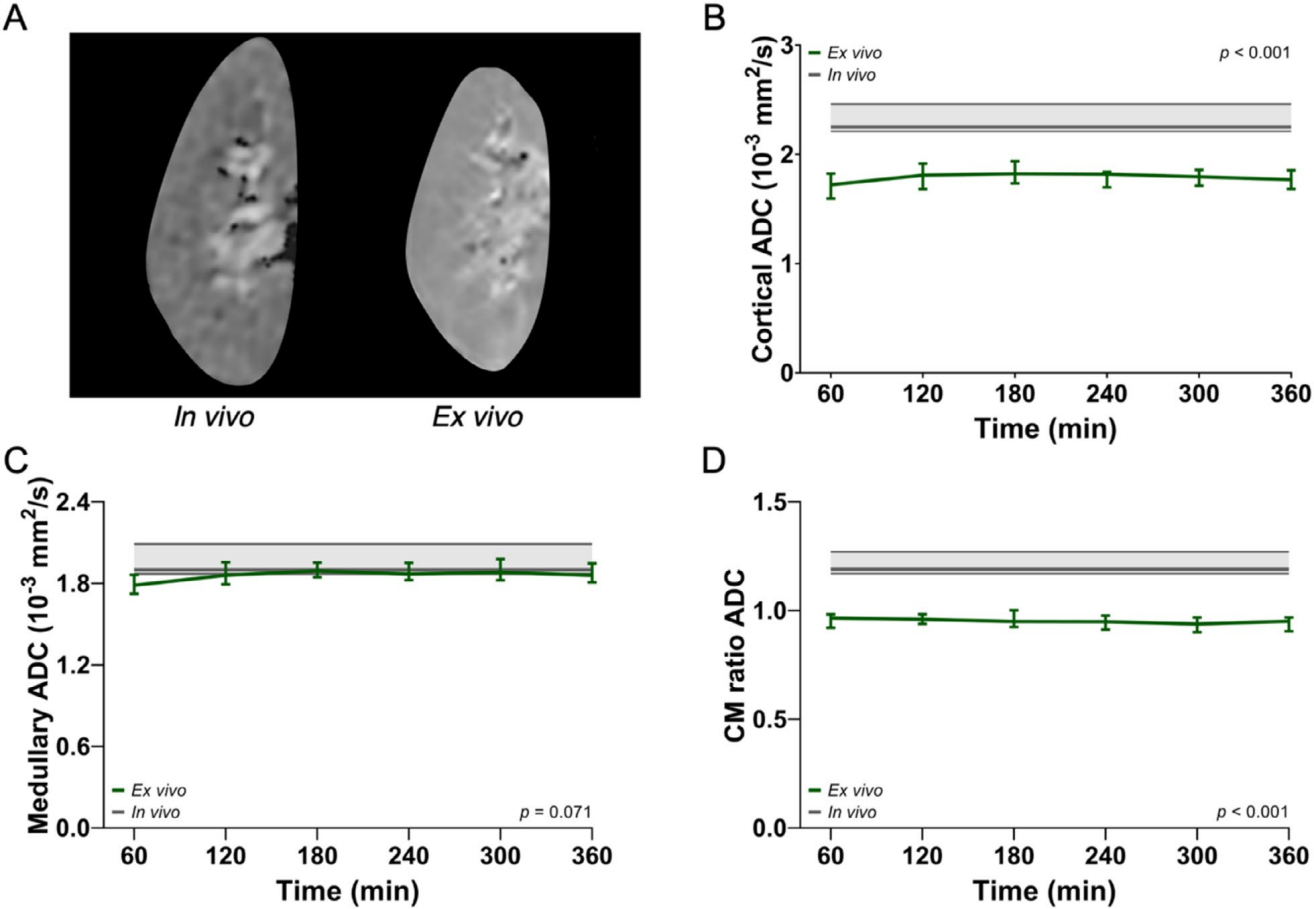
Apparent diffusion coefficient (ADC) maps (A), cortical ADC (B), medullary ADC (C), and corticomedullary ADC ratio (D) of in vivo kidneys from healthy volunteers and ex vivo discarded human kidneys during NMP. Data are presented as median ± IQR. The gray area represents values measured in vivo. ADC, apparent diffusion coefficient; CM, corticomedullary; NMP, normothermic machine perfusion. [Color figure can be viewed at wileyonlinelibrary.com]

The random motion of water molecules can be quantified by diffusion‐weighted imaging (Figure 2A). Low ADC values indicate increased diffusion restriction, which could be associated with tissue edema, vasoconstriction, or interstitial fibrosis [14]. During NMP, cortical ADC values remained relatively stable throughout the 6‐h perfusion period (Figure 2B). Notably, ex vivo kidneys had lower cortical ADC values (1.78 10^−3^ mm^2^/s (IQR 1.68–1.88)) than those in vivo (2.34 10^−3^ mm^2^/s (IQR 2.27–2.37), *p* < 0.001) (Figure 2B). The medullary ADC values were comparable to the in vivo measurements during all hours of NMP (Figure 2C). Consistent with the cortical measurements, the ADC CM ratios were lower in the ex vivo kidneys (0.95 (IQR 0.91–0.98)) compared to the in vivo measurements (1.24 (IQR 1.13–1.32), *p* < 0.001) (Figure 2D).

**FIGURE 3:**
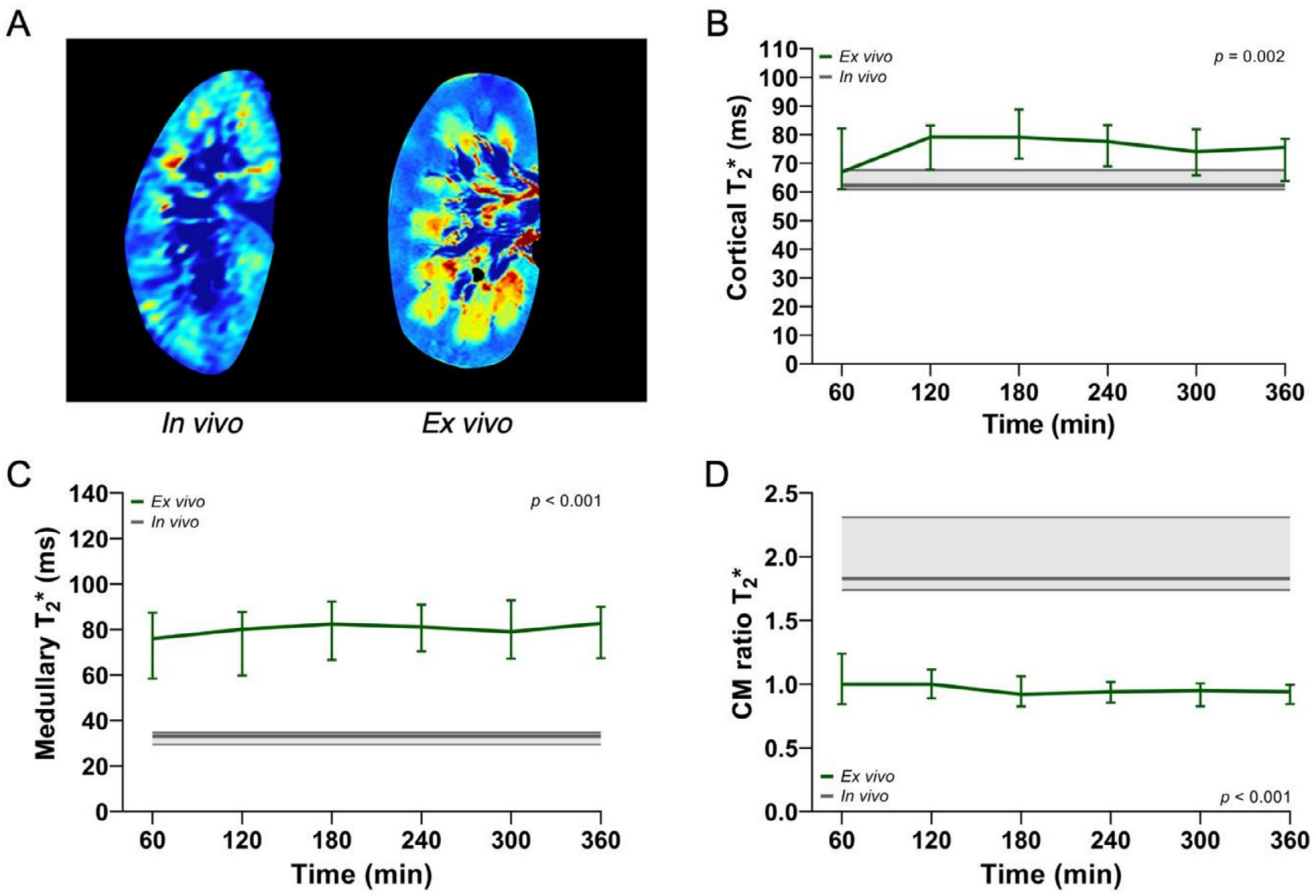
T_2_* maps (A), cortical T_2_* (B), medullary T_2_* (C), and corticomedullary T_2_* ratio (D) of in vivo kidneys from healthy volunteers and ex vivo discarded human kidneys during NMP. Data are presented as median ± IQR. The gray area represents values measured in vivo. CM, corticomedullary; NMP, normothermic machine perfusion. [Color figure can be viewed at wileyonlinelibrary.com]

T_2_* maps were obtained to explore regional differences in oxygen availability within the kidneys (Figure 3A). Higher T_2_* values are typically associated with elevated oxygen availability [15]. Cortical T_2_* values were significantly higher in the ex vivo kidneys (76.3 ms (IQR 64.5–84.2)) compared to the in vivo measurements (61.6 ms (IQR 60.9–64.6), *p* < 0.001) (Figure 3B). Moreover, medullary T_2_* values were also higher in ex vivo kidneys (80.6 ms (IQR 65.3–90.9)) compared to the in vivo measurements (28.1 ms (IQR 26.3–34.8), *p* < 0.001) (Figure 3C). These higher medullary T_2_* values resulted in a higher T_2_* CM ratio in the ex vivo kidneys compared to the in vivo group (Figure 3D).

**FIGURE 4:**
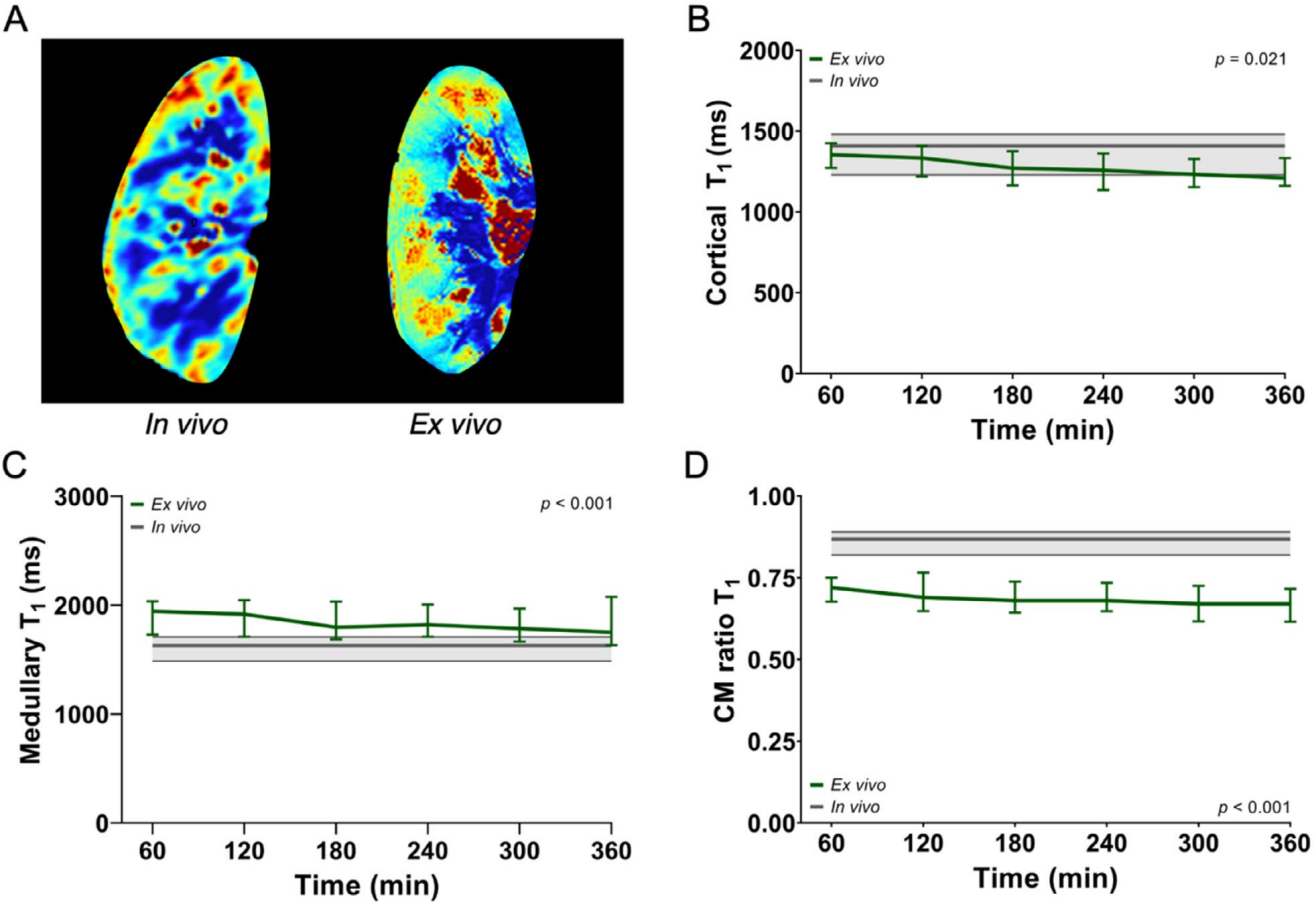
T_1_ maps (A), cortical T_1_ (B), medullary T_1_ (C), and corticomedullary T_1_ ratio (D) of in vivo kidneys from healthy volunteers and ex vivo discarded human kidneys during NMP. Data are presented as median ± IQR. The gray area represents values measured in vivo. CM, corticomedullary; NMP, normothermic machine perfusion. [Color figure can be viewed at wileyonlinelibrary.com]

T_1_ mapping offers quantitative data on pathological processes such as inflammation and fibrosis, and on the water content within the graft [16]. In the discarded ex vivo kidneys, cortical T_1_ values (1272 ms (IQR 1180–1377)) tended to decrease throughout perfusion but were comparable to the healthy in vivo measurements (1318 ms (IQR 1177–1421), *p* = 0.375) (Figure 4B). Moreover, medullary T_1_ values in the ex vivo group (1815 ms (IQR 1685–2036)) were comparable to the in vivo measurements (1614 ms (IQR 1403–1696), *p* = 0.212) (Figure 4C). The calculated CM ratio showed a lower ratio for the ex vivo kidneys compared to the in vivo T_1_ CM ratio (Figure 4D).

**FIGURE 5:**
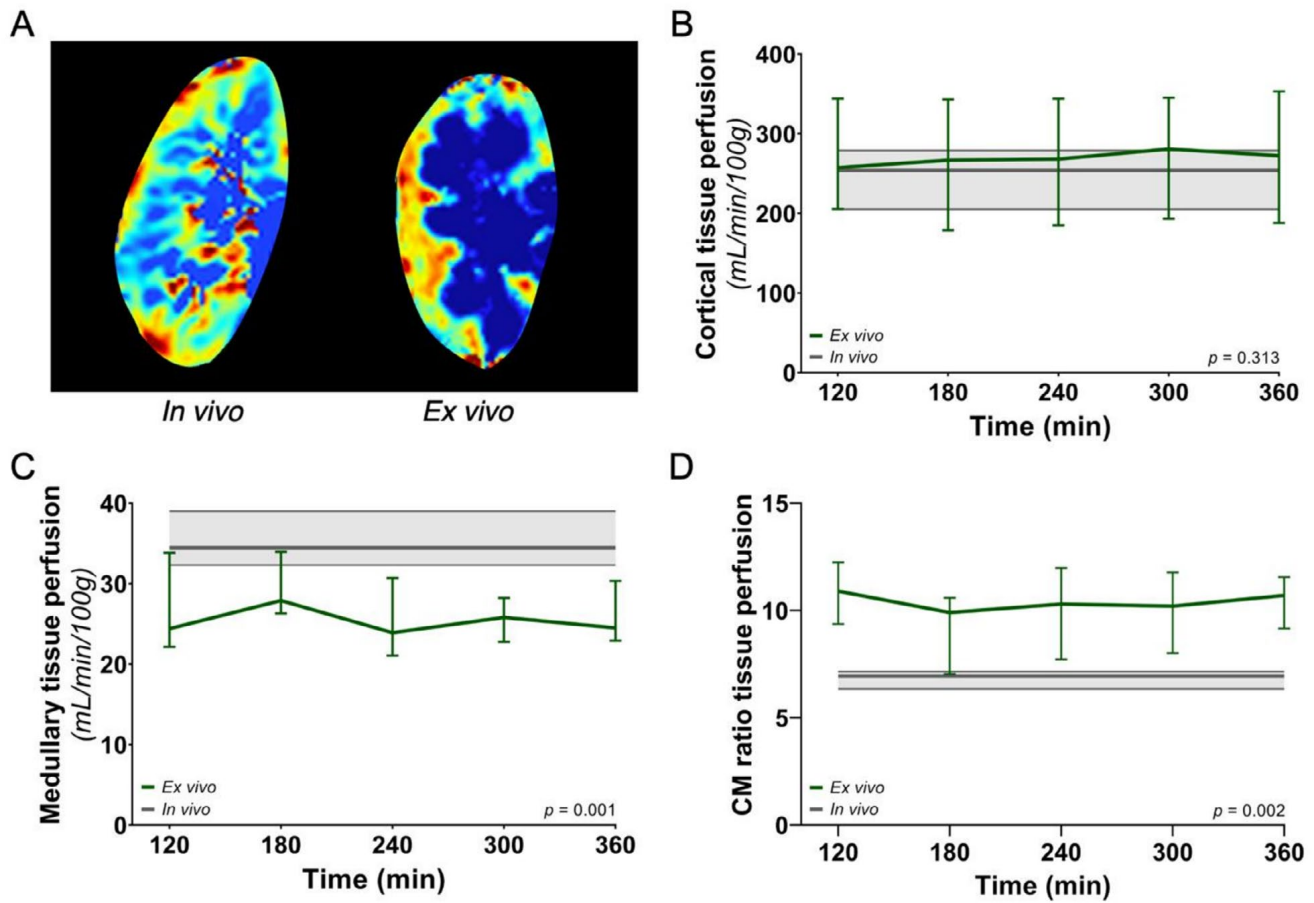
Arterial spin labeling (ASL) maps (A), cortical ASL (B), medullary ASL (C), and corticomedullary ASL ratio (D) of in vivo kidneys from healthy volunteers and ex vivo discarded human kidneys during NMP. Data are presented as median ± IQR. The gray area represents values measured in vivo. CM, corticomedullary; NMP, normothermic machine perfusion. [Color figure can be viewed at wileyonlinelibrary.com]

ASL can non‐invasively quantify renal tissue perfusion, making it a valuable tool for detecting regional perfusion alterations. High ASL values indicate a higher regional tissue perfusion [17]. ASL‐derived cortical tissue perfusion demonstrated considerable variability among all ex vivo kidneys (270 mL/min/100 g; IQR 191–350) and were significantly higher compared to the cortical perfusion of in vivo kidneys from healthy volunteers (215 mL/min/100 g (IQR 197–274), *p =* 0.031) (Figure 5B). The variability was also observed in ex vivo medullary perfusion measurements (26.1 mL/min/100 g (IQR 22.6–32.1)) but was not significantly different compared to in vivo (26.7 mL/min/100 g (IQR 23.8–35.7), *p* = 0.311) (Figure 5C). The variation in both the cortical and medullary measurements resulted in highly variable tissue perfusion CM ratios (10.5 (IQR 7.8–11.7)), ranging between 4.3 and 14.7, and were significantly higher compared to the in vivo CM ratios (7.8 (IQR 7.2–9.2), *p* < 0.001) (Figure 5D).

### 3.3 Donor Characteristics and Ex Vivo Magnetic Resonance Imaging Assessment

**FIGURE 6:**
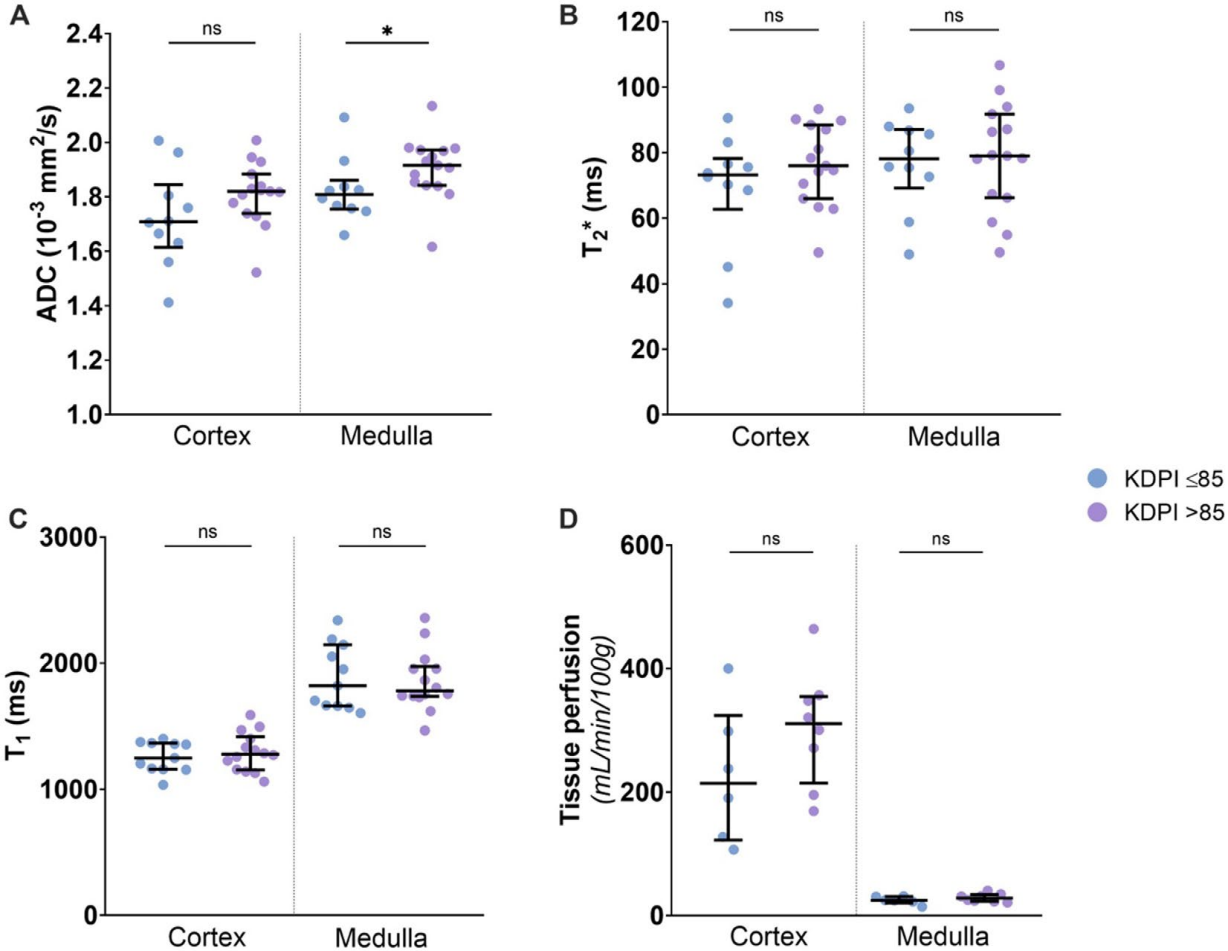
Differences in diffusion‐weighted imaging (A), T_2_* mapping (B), T_1_ mapping (C), and arterial spin labeling (D) of the renal cortex and medulla between kidneys with a low/moderate KDPI (≤ 85) (*n* = 12) and high KDPI (> 85) (*n* = 13). Data are presented as median ± IQR. ADC, apparent diffusion coefficient; KDPI, kidney donor profile index. *p ≤ 0.05; ns, not significant. [Color figure can be viewed at wileyonlinelibrary.com]

To better understand the relationship between the ex vivo acquired MRI data and the donor characteristics, two groups were defined for each donor characteristic (i.e., KDPI, CIT, donor type, donor age, and donor terminal serum creatinine) using either literature‐based or median cutoff values. These groups of ex vivo kidneys were then compared using multiparametric MRI (DWI/ADC, T_2_* mapping, T_1_ mapping, and ASL) to assess potential differences in the renal cortex and medulla. Given the small subgroup sizes, these findings should be viewed as exploratory rather than confirmatory. Since a substantial portion of kidneys with a high KDPI (KDPI score > 85%) are discarded in the USA, this cutoff value was used to define a group with a KDPI score below 85% and a group with a score of 85% and higher. Significantly higher medullary ADC values were observed in the high KDPI group (1.92 × 10^−3^ mm^2^/s (IQR 1.84–1.97)) compared to the group with a KDPI below 85 (1.81 × 10^−3^ mm^2^/s (IQR 1.76–1.86), *p* = 0.027) (Figure 6A). No significant differences were found between the groups in the other cortical and medullary MRI parameters.

**FIGURE 7:**
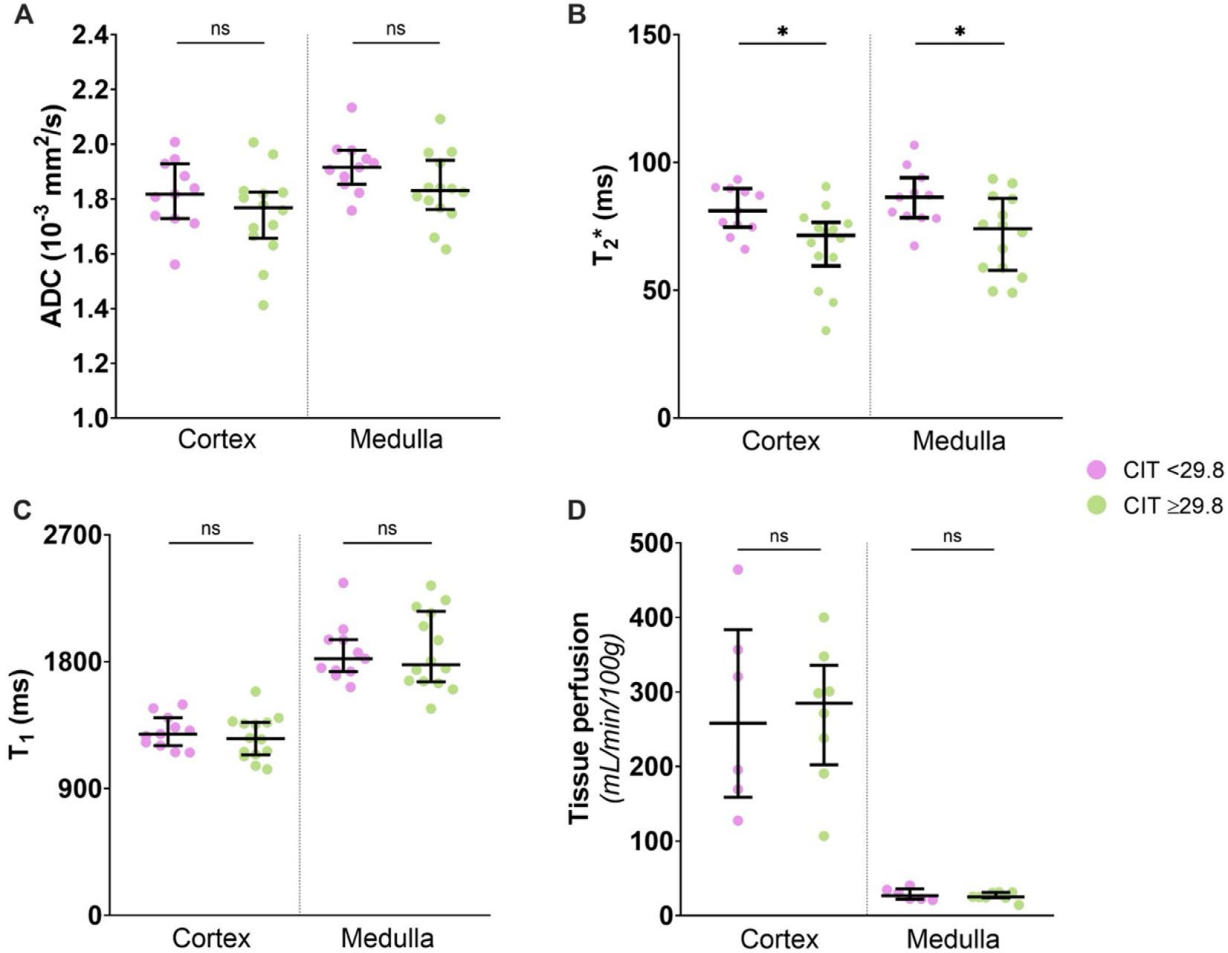
Differences in diffusion‐weighted imaging (A), T_2_* mapping (B), T_1_ mapping (C), and arterial spin labeling (D) of the renal cortex and medulla between kidneys with a CIT duration below (*n* = 11) and above (*n* = 14) 29 h and 50 min. Data are presented as median ± IQR. ADC, apparent diffusion coefficient; CIT, cold ischemia time. *p ≤ 0.05; ns, not significant. [Color figure can be viewed at wileyonlinelibrary.com]

The median CIT duration (29 h and 50 min) was used to divide the discarded kidneys into two groups. Cortical T_2_* values were significantly lower in the group of kidneys with a longer CIT duration (71.5 ms (IQR 59.5–76.6)) compared to the kidneys with a shorter duration (81.1 ms (IQR 74.7–89.8), *p =* 0.015) (Figure 7B). Additionally, the medullary T_2_* values were also significantly lower in the kidneys with a longer CIT duration (71.1 ms (IQR 57.8–85.9)) compared to the group with a short period of CIT (86.4 ms (IQR 78.3–94), *p* = 0.018). There were no significant differences observed in the other MRI modalities.

**FIGURE 8:**
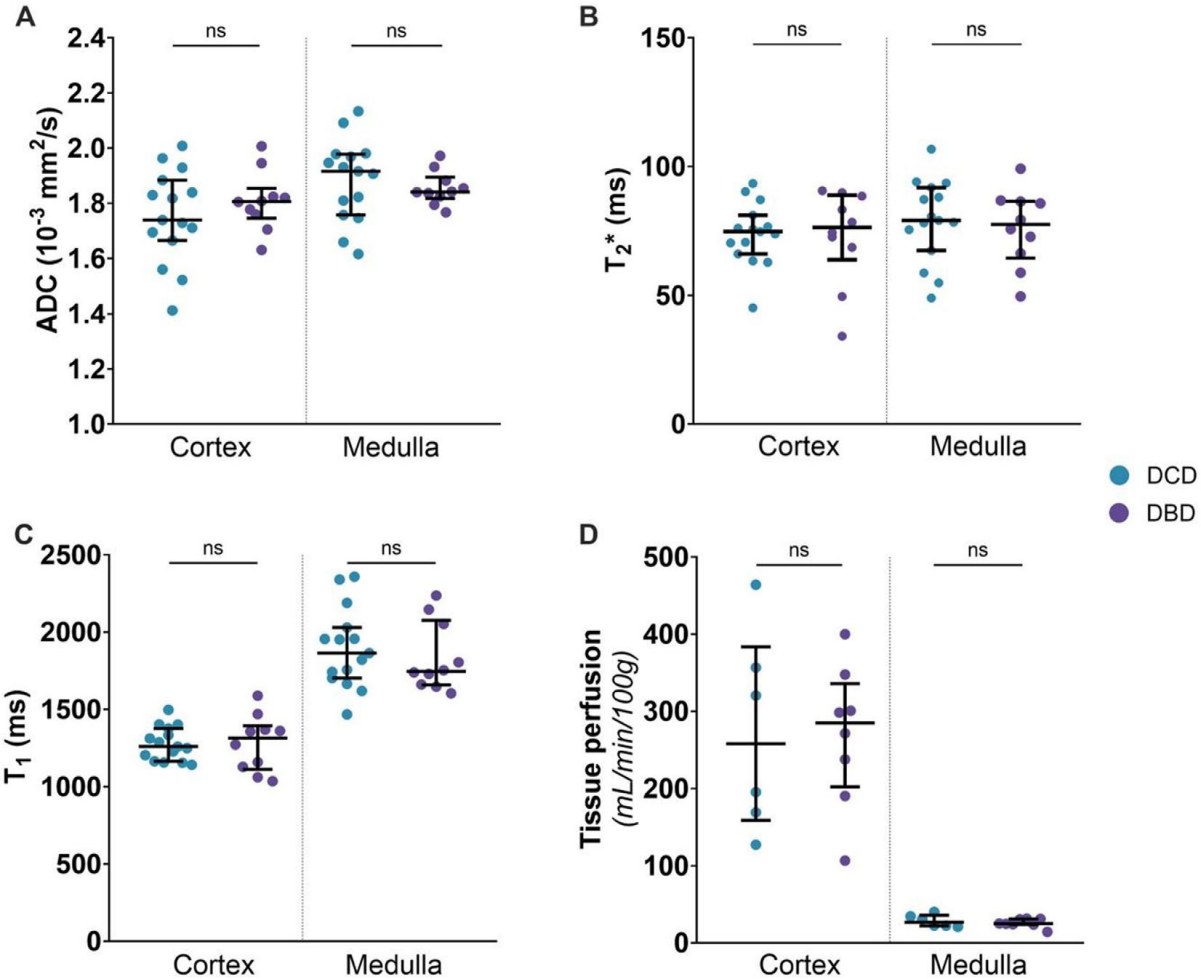
Differences in diffusion‐weighted imaging (A), T_2_* mapping (B), T_1_ mapping (C), and arterial spin labeling (D) of the renal cortex and medulla between kidneys from DCD (*n* = 15) and DBD donors (*n* = 10). Data are presented as median ± IQR. ADC, apparent diffusion coefficient; DBD, donation after brain death; DCD, donation after circulatory death. ns, not significant. [Color figure can be viewed at wileyonlinelibrary.com]

To determine if ischemic injury in DCD grafts could be detected using MRI‐based NMP assessment, a comparison was made between kidneys from DCD and DBD donors. However, no significant differences in MRI‐based characteristics were observed between the kidneys from these two donor types during NMP (Figure 8).

**FIGURE 9:**
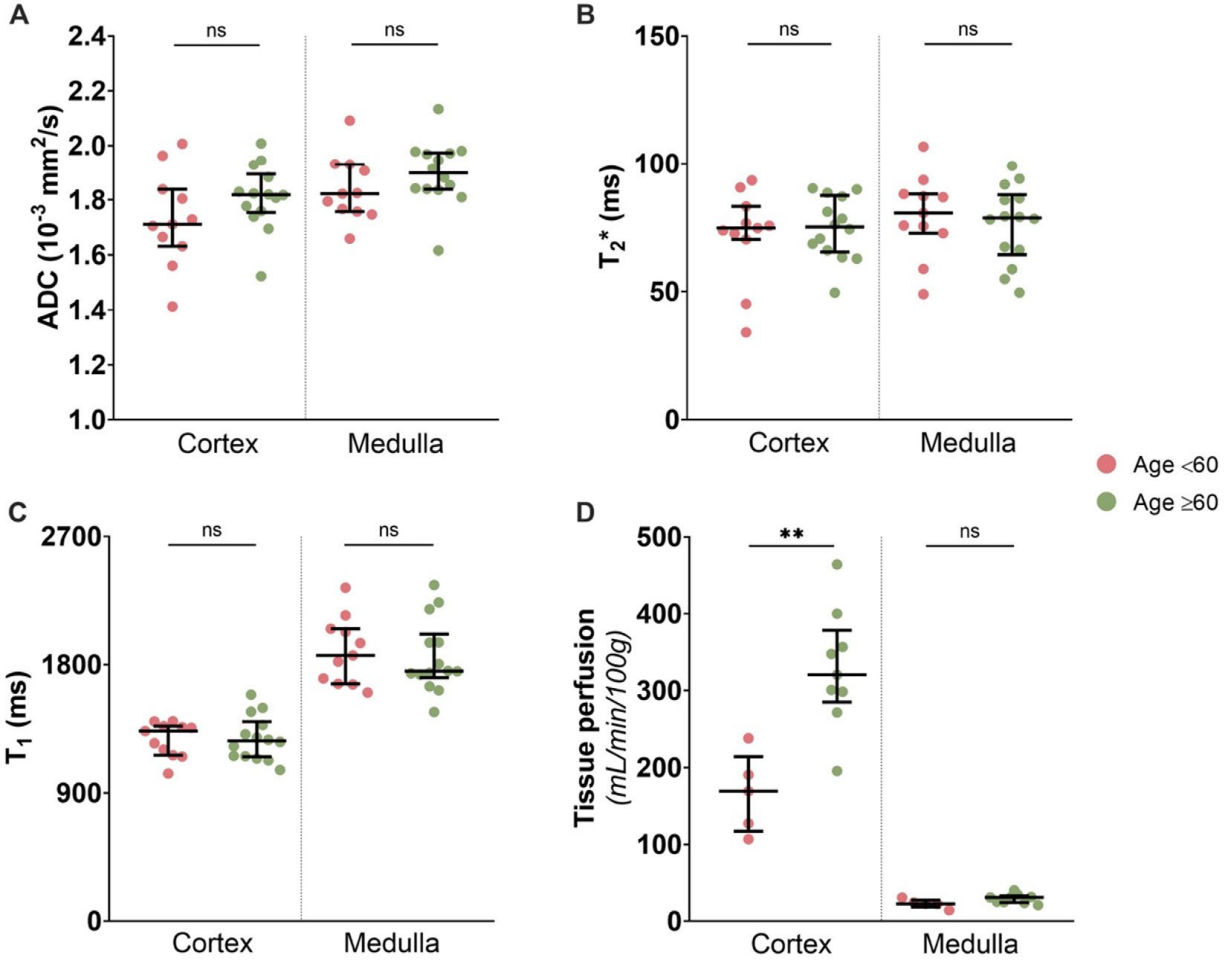
Differences in diffusion‐weighted imaging (A), T_2_* mapping (B), T_1_ mapping (C), and arterial spin labeling (D) of the renal cortex and medulla between kidneys from donors younger than 60 years (*n* = 11) and those aged 60 and above (*n* = 14). Data are presented as median ± IQR. ADC, apparent diffusion coefficient. **p ≤ 0.01; ns, not significant. [Color figure can be viewed at wileyonlinelibrary.com]

Given that kidneys from donors of advanced age are associated with poorer post‐transplant function, two groups were defined between kidneys from donors aged 60 and above and those below 60 years of age. This age threshold represents both the median age in our cohort and the most important cutoff for expanded criteria donors [18]. Our findings revealed a significantly higher cortical tissue perfusion in kidneys from donors aged 60 and above (169 mL/min/100 g (IQR 117–214)) compared to kidneys from donors younger than 60 years (320 mL/min/100 g (IQR 285–378), *p =* 0.002) (Figure 9D).

**FIGURE 10:**
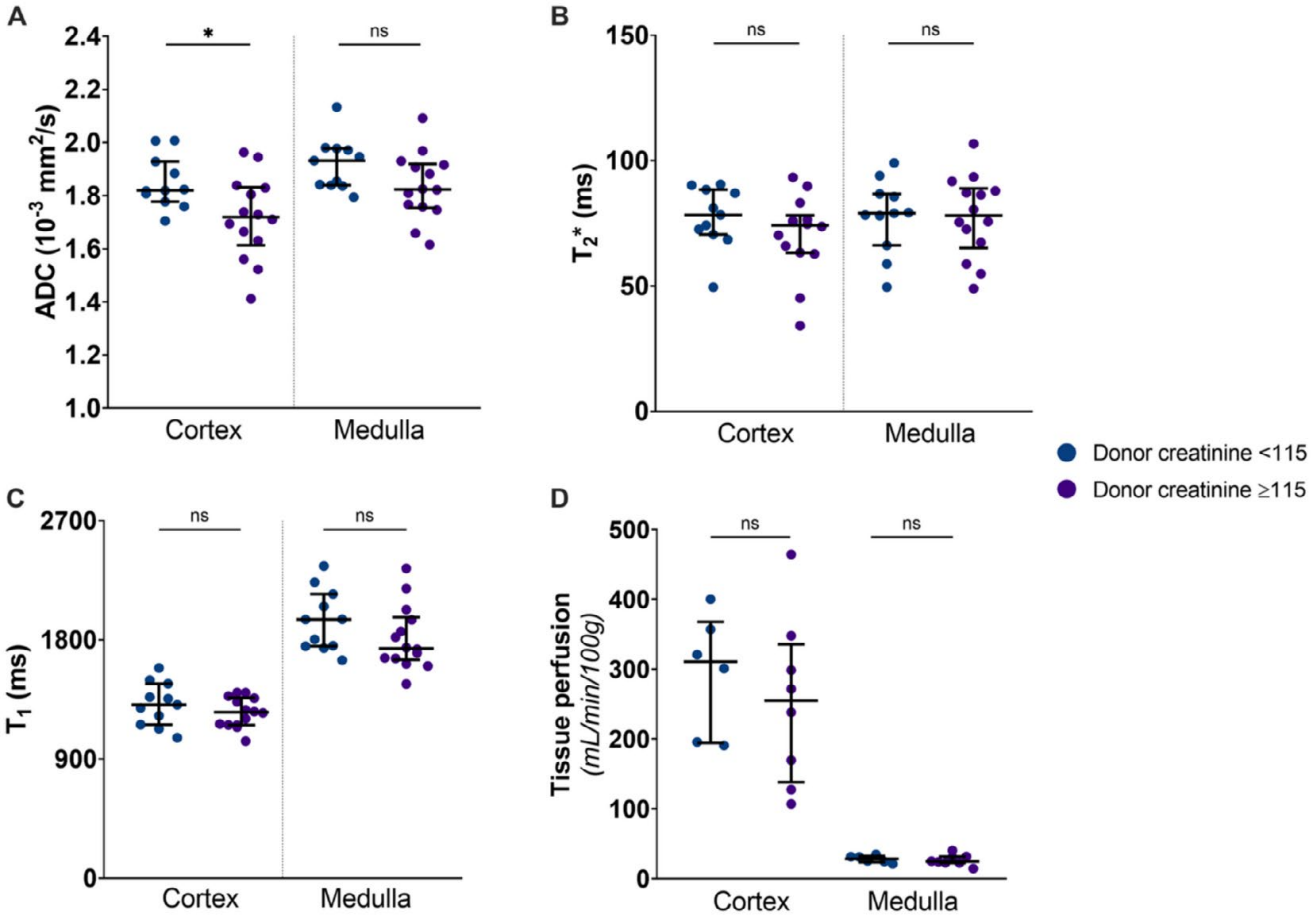
Differences in diffusion‐weighted imaging (A), T_2_* mapping (B), T_1_ mapping (C), and arterial spin labeling (D) of the renal cortex and medulla between kidneys from donors with a terminal serum creatinine below 115 μmol/L and those with a terminal serum creatinine of 115 μmol/L or higher. Data are presented as median ± IQR. ADC, apparent diffusion coefficient. *p ≤ 0.05; ns, not significant. [Color figure can be viewed at wileyonlinelibrary.com]

The groups for the terminal serum creatinine of the donor were defined using a literature‐based cutoff of 115 μmol/L, which represents the upper normal range for adult men [19]. Cortical ADC measurements revealed significantly lower values, indicating increased diffusion restriction, in kidneys from donors with higher terminal serum creatinine levels (1.72 × 10^−3^ mm^2^/s (IQR 1.61–1.83)) compared to those below the cutoff value (1.82 × 10^−3^ mm^2^/s (IQR 1.78–1.93), *p =* 0.033) (Figure 10A). The other measurements showed no significant differences between the groups.

### 3.4 Association Between NMP and Quantitative MRI Parameters

Since the application of MRI during NMP is a novel adjunct assessment strategy, enhancing our understanding of the associations, or potentially even overlap, between specific MRI modalities and conventional NMP parameters is crucial. Therefore, a regression analysis was conducted to investigate such associations (Supporting Information S4). There was a significant positive association between cortical ADC measurements and creatinine clearance during NMP (Supporting Information S4, Figure 1B). Moreover, a higher renal blood flow was associated with a lower cortical T_1_ value (Supporting Information S4, Figure 2A). However, no significant associations were identified between cortical T_2_* values and NMP parameters that could affect or provide insights into oxygen kinetics (e.g., total RBF, CrCl, FENa^+^, VO_2_, ATP content, and lactate concentration). Similarly, no significant associations were found between ASL‐based tissue perfusion and NMP parameters (e.g., total RBF, CrCl, and FENa^+^).

## 4 Discussion

In this study, we present the pioneering use of MRI to investigate differences between ex vivo and in vivo kidney physiology in a human cohort. Furthermore, we also explored whether ex vivo MRI markers can reveal differences in groups based on donor characteristics. Additionally, we examined how conventional NMP parameters relate to multiparametric MRI markers to determine if specific NMP factors influence measured MRI markers. We found that ex vivo kidney MRI markers differ substantially from those in vivo in terms of diffusion, oxygenation, tissue characteristics, and tissue perfusion. However, the comparison with healthy volunteers should be interpreted as a reference framework rather than a direct physiological equivalent, since the observed differences likely reflect both NMP‐related perfusion effects and underlying donor pathology. Moreover, while some donor‐related differences and associations with NMP parameters were observed, most MRI measurements appeared largely independent of these factors, suggesting they may offer complementary information about donor graft characteristics.

MRI biomarkers have shown promise in identifying acute and chronic kidney disease, but logistical and technical challenges have limited their broader application in research and clinical practice [20]. Building on our previous porcine MRI study [12], we aimed to validate these findings in human kidneys to better reflect clinical realities. The porcine study revealed greater diffusion restriction in ex vivo kidneys compared to in vivo. Consistent with these findings, discarded human donor kidneys also exhibited diffusion restriction during NMP compared to the in vivo measurements of healthy individuals. Decreased ADC values, indicative of increased restriction, have been found in kidneys with impaired post‐transplant function [21]. Moreover, DWI has been demonstrated to detect chronic kidney disease and correlated with estimated glomerular filtration rate [22]. We also demonstrated that cortical ADC values were significantly higher in kidneys obtained from donors with a terminal serum creatinine below 115 μmol/L compared to those from donors with higher serum creatinine levels. Importantly, cortical ADC was the MRI parameter most closely associated with functional output during NMP, demonstrating a significant positive relationship with creatinine clearance. These findings indicate that DWI may have potential as a complementary tool for assessing graft function during NMP, although further validation in a transplantation model is required.

BOLD‐MRI/T_2_*‐mapping exploits the paramagnetic properties of deoxygenated hemoglobin. Decreased oxygenation increases deoxyhemoglobin levels, leading to a decrease in T_2_* signal, providing insights into regional oxygen availability. It has been demonstrated that advancing stages of chronic kidney disease are associated with increased medullary T_2_* values, while cortical T_2_* values decreased [23]. In our porcine study, we observed higher medullary T_2_* values in ex vivo kidneys compared to in vivo. This trend was also evident in the human cohort, where medullary values of the ex vivo kidneys were higher than the in vivo measurements. In addition, cortical values during NMP of discarded human ex vivo kidneys exhibited a great variability, with values both above and below the in vivo range. The observed variability in cortical and medullary measurements among discarded human kidneys may reflect differences in regional oxygen handling during NMP. In particular, medullary measurements might relate to tubular metabolic activity, such as oxygen‐dependent solute reabsorption, and could therefore provide additional insight into tissue function during perfusion but also requires further investigation in a transplantation model.

T_1_ mapping is used to evaluate renal pathologies such as inflammation and fibrosis, and water content within the parenchyma. In normal physiology, and our in vivo measurements, the medullary T_1_ value is slightly higher compared to the cortex, which is attributed to a higher free water content in the medullary tubules and collecting ducts [24]. Reduced CMDs and increased T_1_ values in both cortex and medulla have been reported in kidneys with acute and chronic allograft rejection and acute kidney injury [25, 26, 27]. In our study, ex vivo cortical and medullary T_1_ values were comparable to in vivo measurements, questioning their relevance for graft quality assessment during NMP.

To explore flow distribution in ex vivo kidneys, we used ASL, a non‐invasive method to quantify renal tissue perfusion. By tagging protons in inflowing arterial blood (or NMP perfusate) and measuring them after a delay, ASL generates perfusion maps without contrast agents [17]. An ideal RBF distribution allocates ~85% to the cortex and 15% to the medulla, crucial for maintaining the osmotic gradient required for urine concentration [28, 29]. During ex vivo perfusion of the discarded human kidneys, a wide range of CM ratios was observed, while the in vivo CM ratio of healthy kidneys was approximately 8. Furthermore, the ASL‐derived perfusion showed no association with the total RBF measured with a flow sensor on the arterial inflow line. The lack of association between ASL‐derived and total NMP blood flow was observed before by our group [9], and could be partially attributed to the high variability in total RBF between the kidneys during NMP, ranging from 40 to 500 mL/min/100 g. Since ASL is measured with a set delay, the high variability in total RBF can affect the measured ASL signal when flow exceeds the set delay. Since the total RBF was within a narrower range in the porcine experiments, ASL perfusion measurements were more consistent in this study. This highlights the challenge of accurately measuring ASL in kidneys with highly fluctuating perfusion rates.

This study had several limitations. While CITs are typically longer in the USA than in Europe, the discarded human kidneys from OPOs across the USA used in this study experienced even longer CITs than usual US clinical preservation times due to extended transportation durations. Although prolonged CIT is often associated with poorer post‐transplant function, research has shown that extended CITs only have a modest impact on kidney viability after transplantation [30, 31], indicating that the kidneys used in this study still retain functional potential. Furthermore, the variability in donor characteristics and CIT duration, combined with the decision not to predefine groups prior to the start of the study, led to high data variability. Nevertheless, this approach closely resembles the diversity found in clinical kidneys available for transplantation and provides an opportunity to explore the relationship between ex vivo MRI characteristics and conventional NMP measurements. Despite collecting data on DWI, T2*‐mapping, and T1 mapping from all 25 experiments, ASL data could only be obtained from 15 experiments due to a delay in the approval of the controller‐to‐processor agreement for the pCASL sequence. Moreover, a key limitation is that, in the setting of highly variable perfusion rates during NMP, ASL measurements are best regarded as semi‐quantitative. The use of a fixed post‐labeling delay, while necessary for protocol consistency, may introduce inaccuracies when flow rates exceed the assumed transit time, thereby affecting perfusion estimates.

Most importantly, this study did not yet incorporate actual transplantation of ex vivo perfused kidneys and therefore lacks important outcome data, which could confirm or disprove the suggested added diagnostic value of MRI measurements during NMP.

In conclusion, after characterizing differences between ex vivo and in vivo physiology in a porcine model, this study provides the first evidence that similar differences occur in ex vivo perfused human donor kidneys compared with healthy volunteers. These findings are specific to a high‐risk, discarded donor population. While the added value of MRI during clinical NMP remains unclear, the limited associations with conventional NMP parameters and the variability in MRI measurements suggest that it could offer complementary insights into graft viability. Integration of MRI with conventional perfusion metrics and future multi‐omics approaches may ultimately enable the development of a more comprehensive set of biomarkers for assessing graft viability.

## Author Contributions

T.L.H., C.L.J., N.O., V.A.L., X.Z., U.D., R.J.H.B., H.G.D.L., A.K.K., and C.M. conceptualized the research design. T.L.H., C.L., V.A.L., B.O., and J.C. participated in the performance of the research. T.L.H. and L.A.v.F. drafted the manuscript. C.L.J., J.C., N.O., V.A.L., B.O., X.Z., U.D., R.J.H.B., H.G.D.L., A.K.K., and C.M. performed the critical revision.

## Funding

This work was supported by the European Research Council (ERC) under the European Union's Horizon 2020 research and innovation program (Grant agreement No. 851368, C.M.). This project was partially funded by the Indiana Clinical and Translational Sciences Institute through Grant Number UM1TR004402 from the National Institutes of Health, National Center for Advancing Translational Sciences, as part of the Clinical and Translational Sciences Award.

## Conflicts of Interest

C.L.J. is co‐founder and CEO of 34Lives, H.G.D.L. is part‐time CSO of 34Lives. Other authors declare no conflicts of interest.

## Supporting information


**Figure S1:** Association of cortical ADC values with renal blood flow (A), creatinine clearance (B), fractional sodium excretion (C), and renal weight gain during NMP (D). Data are represented as mean values of the 6 h of NMP. The models were adjusted for KDPI. KDPI; kidney donor profile index. *p ≤ 0.05.
**Figure S2:** Association of cortical T_1_ values with renal blood flow (A), creatinine clearance (B), fractional sodium excretion (C), and renal weight gain during NMP (D). Data are represented as mean values of the 6 h of NMP. The models were adjusted for KDPI. FE_Na_^+^, fractional sodium excretion; KDPI; kidney donor profile index. *p ≤ 0.05.
**Figure S3:** Association of cortical T_2_* values with renal blood flow (A), creatinine clearance (B), fractional sodium excretion (C), oxygen consumption (D), adenosine triphosphate content (E), and lactate (F). Data are represented as mean values of the 6 h of NMP. The models were adjusted for KDPI. ATP, adenosine triphosphatase; FE_Na_^+^, fractional sodium excretion; KDPI; kidney donor profile index; VO_2_, oxygen consumption.
**Figure S4:** Association of cortical arterial spin labeling with renal blood flow (A), creatinine clearance (B), and fractional sodium excretion (C). Data are represented as mean values of the 6 h of NMP. The models were adjusted for KDPI. FE_Na_^+^, fractional sodium excretion; KDPI; kidney donor profile index.

## Data Availability

Data generated or analyzed during this study are available from the corresponding author upon reasonable request.
