# Supplementary material for: Magnetic Resonance Imaging Evaluation of Functional Differences Between In Vivo Human Kidneys and Discarded Human Kidneys During Ex Vivo Normothermic Machine Perfusion
Source: Artif Organs. 2026 Apr 27;50(7):1037–51. doi: 10.1111/aor.70142 (PMC13397312; doi:10.1111/aor.70142)
Supplement: Supplementary file 1 — Figure S1: Association of cortical ADC values with renal blood flow (A), creatinine clearance (B), fractional sodium excretion (C), and renal weight gain during NMP (D). Data are represented as mean values of the 6 h of NMP. The models were adjusted for KDPI. KDPI; kidney donor profile index. *p ≤ 0.05. Figure S2: Association of cortical T1 values with renal blood flow (A), creatinine clearance (B), fractional sodium excretion (C), and renal weight gain during NMP (D). Data are represented as mean values of the 6 h of NMP. The models were adjusted for KDPI. FENa +, fractional sodium excretion; KDPI; kidney donor profile index. *p ≤ 0.05. Figure S3: Association of cortical T2* values with renal blood flow (A), creatinine clearance (B), fractional sodium excretion (C), oxygen consumption (D), adenosine triphosphate content (E), and lactate (F). Data are represented as mean values of the 6 h of NMP. The models were adjusted for KDPI. ATP, adenosine triphosphatase; FENa +, fractional sodium excretion; KDPI; kidney donor profile index; VO2, oxygen consumption. Figure S4: Association of cortical arterial spin labeling with renal blood flow (A), creatinine clearance (B), and fractional sodium excretion (C). Data are represented as mean values of the 6 h of NMP. The models were adjusted for KDPI. FENa +, fractional sodium excretion; KDPI; kidney donor profile index. [file AOR-50-1037-s001.docx]

**Supplementary materials**

**Supplementary material 1**

| **Perfusate constituents** | | | | | |  |
| --- | --- | --- | --- | --- | --- | --- |
| **Constituent** |  | | | | *Total circulating volume: 1250 ml* |  |
|  |  |  |  |  |  |  |
| **Washed red blood cells**  Including NaCl 0.9% (Fresenius Kabi Nederland B.V. Zeist, the Netherlands | | |  | | Total weight 850 g |  |
| **Sterile water**  (Fresenius Kabi) | | |  | | 226 mL |  |
| **Human albumin 200 g/L**  (Sanquin Plasma Products B.V., Amsterdam, the Netherlands) | | |  | | 200 mL |  |
| **NaHCO_3_ 8.4%** (B. Braun Melsungen AG, Melsungen, Germany) | | |  | | 40 mL |  |
| **Calcium gluconate 10%** (B. Braun) | | |  | | 8 mL |  |
| **Mannitol 25%** (Fresenius Kabi) | | |  | | 6 mL |  |
| **Cefazolin 1 g**  (Sandoz B.V. Almere, the Netherlands) **dissolved in 20 mL NaCl 0.9%** (Fresenius Kabi) | | |  | | 20 mL |  |
| **Aminoplasmal 10%**  (B. Braun) | | |  | | 10 mL |  |
| **Glucose 25%**  (Pfizer Inc., New York, NY, USA) | | |  | | 5.6 mL |  |
| **MgSO_4_ 100 mg/mL** (Teva Nederland B.V., Haarlem, the Netherlands) | | |  | | 1 mL |  |
| **Na_3_PO_4_ 3 mmol/mL** (Apotheek A15, Gorinchem, the Netherlands) | | |  | | 0.3 mL |  |
| **Cernevit** (Baxter B.V.) **dissolved in 5 mL sterile water** (Fresenius Kabi) | | |  | | 1 mL |  |
| **Heparin 25000 IU/mL**  (Sagent Pharmaceuticals, Schaumburg, IL, USA) | | |  | | 1 mL |  |
| **Creatinine**  (Sigma-Aldrich, Zwijndrecht, the Netherlands) | | |  | | 0.115 g |  |
| **Infusion pump 1 (3 mL/h)** | | | | | | |
| **Flolan 0.5 mg**  (GlaxoSmithKline, Middlesex, United Kingdom)  **dissolved in 50 ml solvent of which 6 mL was added to 39 mL of NaCl 0.9%** (Fresenius Kabi) | | **OR** | | **Verapamil 2.5 mg/mL**  (Centrafarm B.V., Breda, the Netherlands) **4 mL dissolved in 36 mL of NaCl** (Fresenius Kabi) | | |
| **Infusion pump 2 (3 mL/h)** | | | | | | |
| **Glucose 25%**  (Baxter B.V.) **6 mL dissolved in 24 mL sterile water** (Fresenius Kabi) | | |  | |  |  |

**Supplementary material 2**

**Creatinine clearance:**

CrCl (mL/min/100g) = $\left( \frac{\left( \frac{U_{creatinine}}{P_{creatinine}} \right) * diuresis}{g} \right)*100$

Urinary and perfusate creatinine concentration in mmol/L, diuresis in mL/min.

**Fractional excretion of sodium:**

FE_Na_^+^ (%) = $\left( \frac{U_{Na}* P_{creatinine}}{P_{Na}* U_{creatinine}} \right)*100$

Urinary and perfusate sodium concentration in mmol/L, perfusate and urinary creatinine concentration in µmol/L.

**Formula for oxygen consumption during NMP:**

VO_2_ (mLO_2_/min/100g) = $\left( \frac{\left( \left( Hb * 2.4794 \right)+\left( {pO}_{2 arterial} * K \right) \right)-\left( \left( 0.024794 * Hb * {sO}_{2 venous} \right)+\left( {pO}_{2 venous}* K \right) \right)*Q}{g} \right)*100$

Where Hb is the hemoglobin concentration in mmol/L, pO_2_ is the partial oxygen pressure in kPa, K is the solubility constant of oxygen in water at 37°C which equals 0.0225 (mL O_2_ per kPa), sO_2_ is the saturation in %, Q is the renal perfusate flow (dL/min), renal weight before NMP in grams.

**Supplementary materials 3**

| Parameters of MRI sequences | | | | | | | | | | | |  |
| --- | --- | --- | --- | --- | --- | --- | --- | --- | --- | --- | --- | --- |
| Sequence |  | **T_2w_** |  | **ASL** |  | **BOLD/T_2_* map** |  | **DWI/ADC map** |  | **T_1_ map** | |  |
| Unit |  | SI |  | mL/min/100g |  | ms |  | 10^-3^ mm^2^/s |  | ms | |  |
| Type |  | Fast Spin Echo,  Echo Train Length 21 |  | pCASL  Multi-PLD |  | Fast Gradient Echo / Multi-Echo Sequence |  | Resolve, b-values = 0, 500, 1000 s/mm^2^ |  | Multi-echo, saturation-recovery, Turbo Spin-Echo | |  |
| 2D or 3D |  | 2D |  | 2D |  | 2D |  | 2D |  | 2D | |  |
| Acquisition plane |  | Coronal |  | Coronal |  | Coronal |  | Coronal |  | Coronal | |  |
| Repetition time (ms) |  | 1010 |  | 5000 |  | 1000 |  | 2110 |  | 4350 | |  |
| Echo time (ms) |  | 133 |  | 16 |  | 7 echo times, 4 – 19 |  | 68, 102 |  | 23, 101 | |  |
| Flip angle (°) |  | 100 (refocusing flip angle) |  | 90 |  | 60 |  | 180  (refocusing flip angle) |  | | 150 |  |
| Number of slices |  | 144 |  | 31 |  | 20 |  | 15 |  | 15 | |  |
| Slice thickness (mm) |  | 0.7 |  | 4.5 |  | 4 |  | 4 |  | 4 | |  |
| Voxel size (mm) |  | 0.5 x 0.5 x 0.7 |  | 1.6 x 1.6 x 4.5 |  | 0.9 x 0.9 x 4.0 |  | 1.9 x 1.9 x 4.0 |  | 0.8 x 0.8 x 4.0 | |  |
| Bolus duration (ms) |  | n/a |  | 700 |  | n/a |  | n/a |  | n/a | |  |
| Post labeling delay (ms) |  | n/a |  | 100, 300, 500 |  | n/a |  | n/a |  | n/a | |  |
| Bandwidth (Hz/pixel) |  | 248 |  | 1954 |  | 260 |  | 930 |  | 150 | |  |
| Total acquisition time (min) |  | 6:34 |  | 5:37 |  | 5:48 |  | 5:30 |  | 6:07 | |  |
| ADC, apparent diffusion coefficient; ASL, arterial spin labeling; BOLD, blood oxygen level-dependent; DWI, diffusion-weighted imaging; pCASL, pseudo-continuous arterial spin labeling; PLD, post-labeling delay.  SI, signal intensity; n/a, not applicable. | | | | | | | | | | | | |

**Supplementary materials 4**


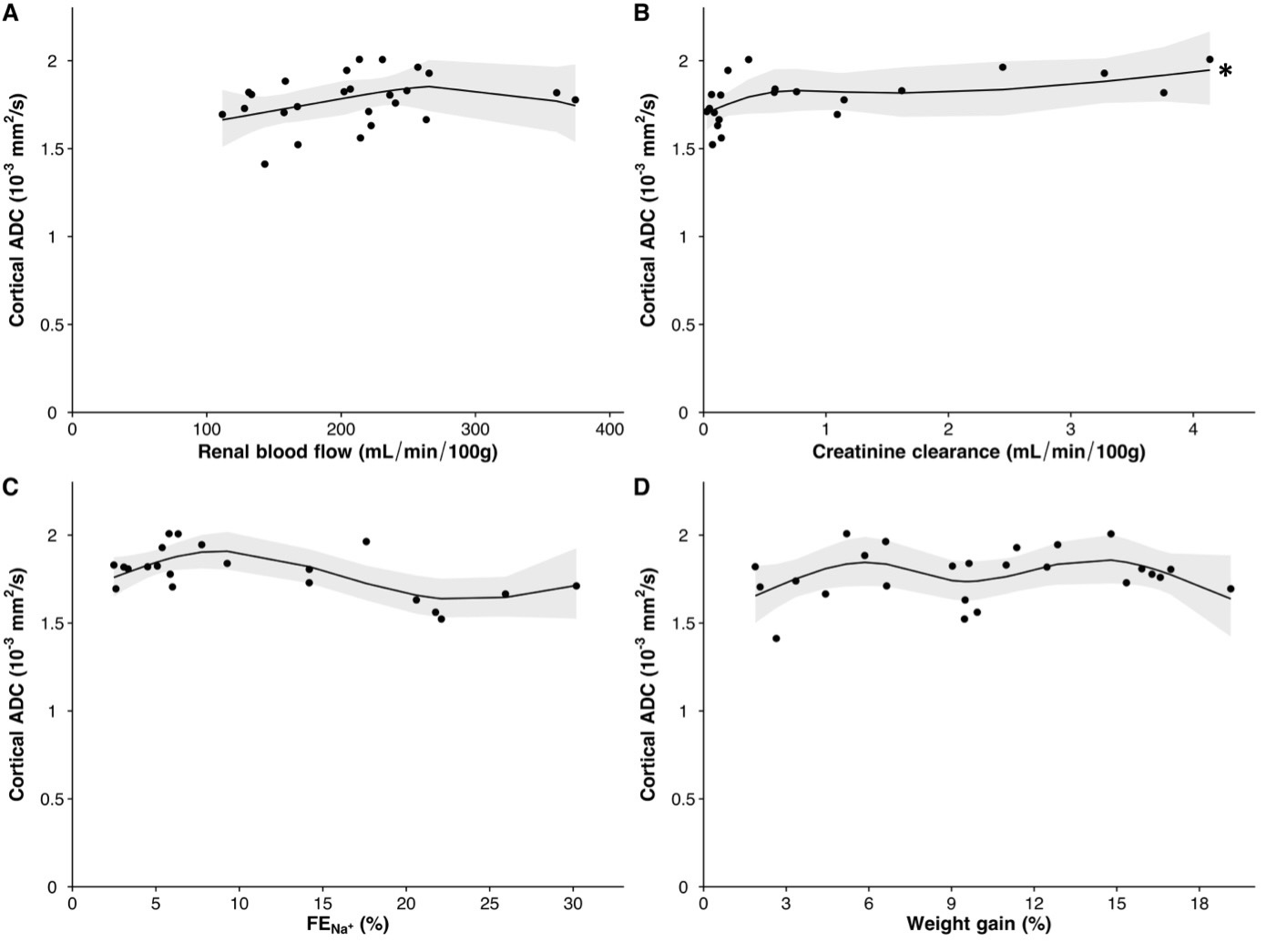


**Figure 1. Association of cortical ADC values with renal blood flow (A), creatinine clearance (B), fractional sodium excretion (C), and renal weight gain during NMP (D).** Data are represented as mean values of the 6 hours of NMP. The models were adjusted for KDPI. KDPI; kidney donor profile index. **p* ≤ 0.05.

**
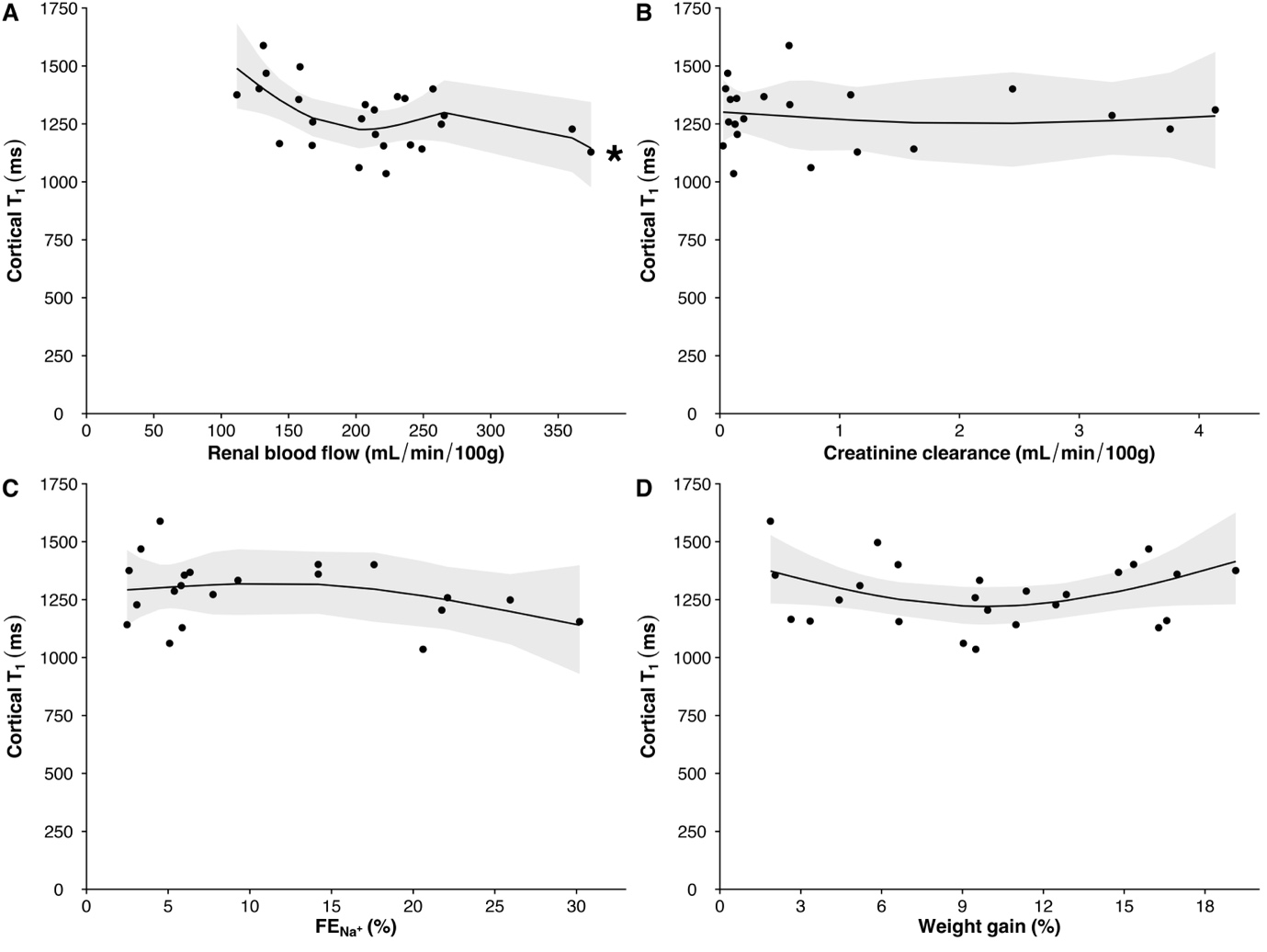
Figure 2. Association of cortical T_1_ values with renal blood flow (A), creatinine clearance (B), fractional sodium excretion (C), and renal weight gain during NMP (D).** Data are represented as mean values of the 6 hours of NMP. The models were adjusted for KDPI. FE_Na_^+^, fractional sodium excretion; KDPI; kidney donor profile index. **p* ≤ 0.05.


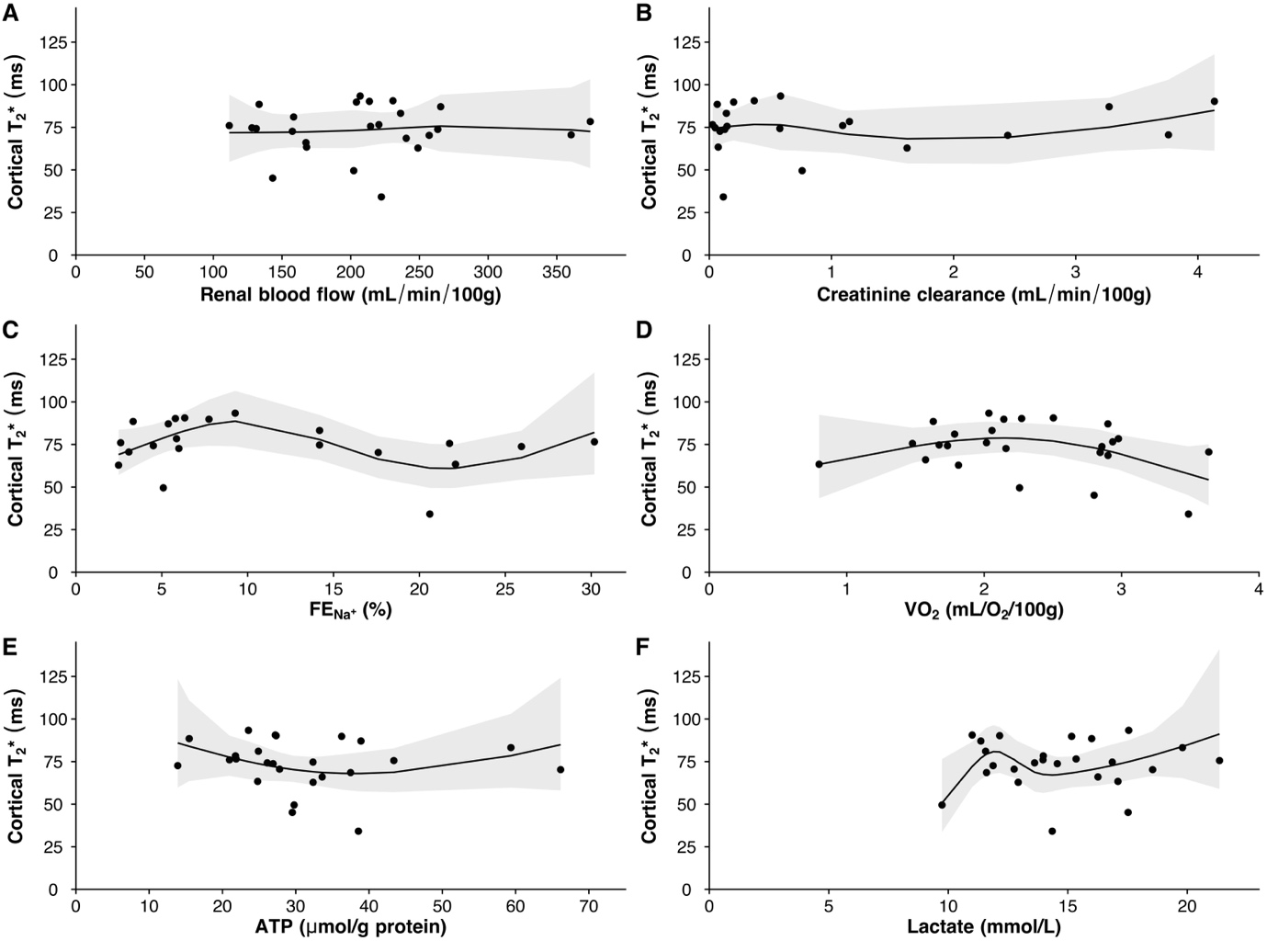


**Figure 3. Association of cortical T_2_* values with renal blood flow (A), creatinine clearance (B), fractional sodium excretion (C), oxygen consumption (D), adenosine triphosphate content (E), and lactate (F).** Data are represented as mean values of the 6 hours of NMP. The models were adjusted for KDPI. ATP, adenosine triphosphatase; FE_Na_^+^, fractional sodium excretion; KDPI; kidney donor profile index; VO_2_, oxygen consumption.

**
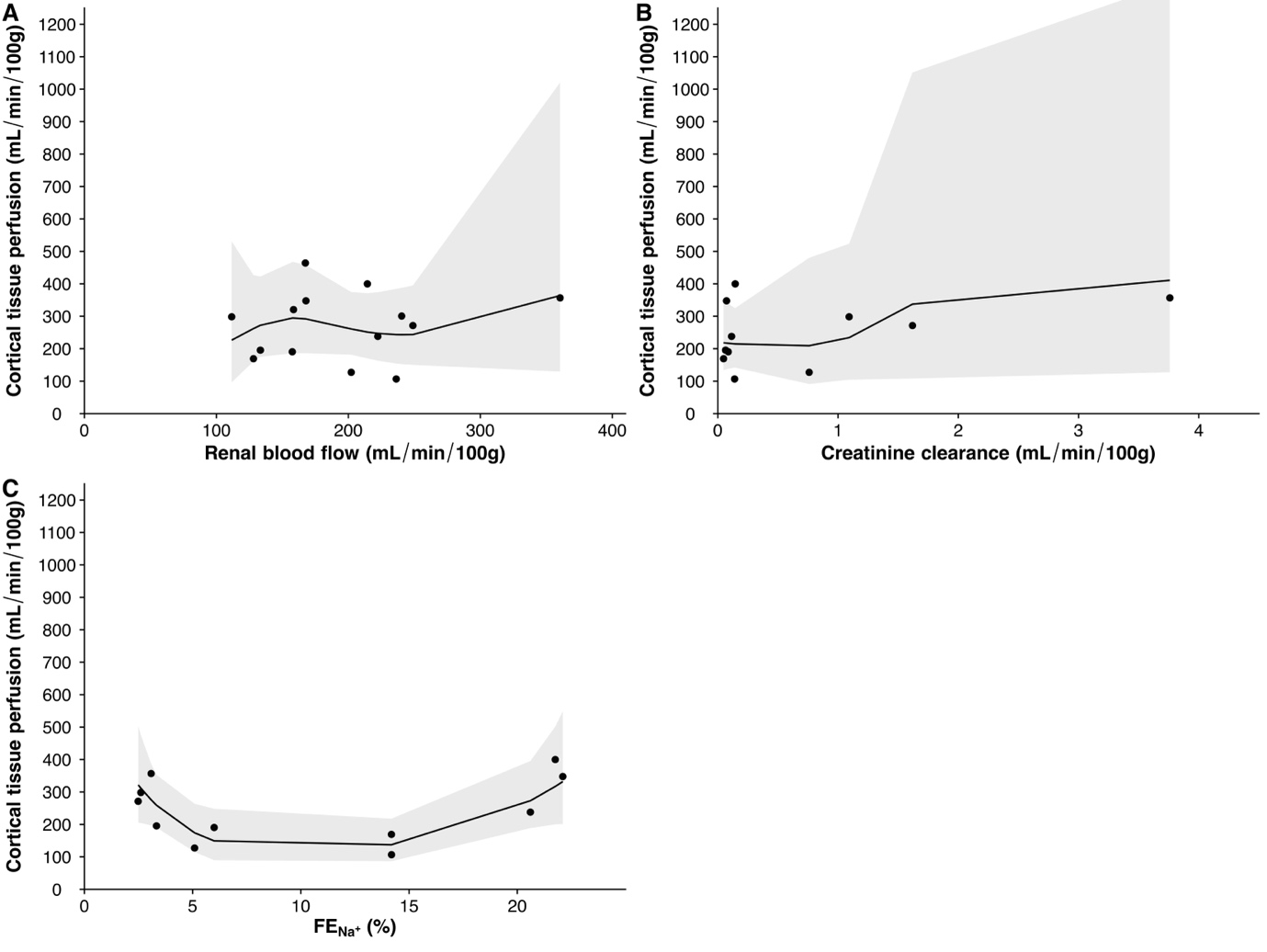
**

**Figure 4. Association of cortical arterial spin labeling with renal blood flow (A), creatinine clearance (B), and fractional sodium excretion (C).** Data are represented as mean values of the 6 hours of NMP. The models were adjusted for KDPI. FE_Na_^+^, fractional sodium excretion; KDPI; kidney donor profile index.
